# Diagnostic Bioliquid Markers for Pancreatic Cancer: What We Have vs. What We Need

**DOI:** 10.3390/cancers15092446

**Published:** 2023-04-25

**Authors:** Geou-Yarh Liou, Crystal J. Byrd

**Affiliations:** 1 Center for Cancer Research and Therapeutic Development, Clark Atlanta University, Atlanta, GA 30314, USA; 2 Department of Biological Sciences, Clark Atlanta University, Atlanta, GA 30314, USA

**Keywords:** pancreatic ductal adenocarcinoma, biomarker, early detection, early diagnosis, resectable pancreatic cancer, secreted factors, liquid biopsy, exosomes

## Abstract

**Simple Summary:**

Among all types of pancreatic cancer, pancreatic ductal adenocarcinoma (PDAC) is the most common type and has an extremely low survival rate. Early detection at an early stage, when surgical removal is still available, is crucial to minimize the death toll of the individuals who are dying from PDAC. In this review, we have summarized the liquid biomarkers that are currently being used to diagnose PDAC in the clinic, clinical trials, and under development for potential use in the future. This review also provides insight into future liquid biomarkers that may be used in routine examinations for the early diagnosis of PDAC development and its precursors, hoping to significantly decrease PDAC death numbers.

**Abstract:**

Pancreatic ductal adenocarcinoma (PDAC), the most common type of pancreatic cancer, currently has a dismal five-year survival rate of approximately 10% due to late diagnosis and a lack of efficient treatment options such as surgery. Furthermore, the majority of PDAC patients have surgically unresectable cancer, meaning cancer cells have either reached the surrounding blood vessels or metastasized to other organs distant from the pancreas area, resulting in low survival rates as compared to other types of cancers. In contrast, the five-year survival rate of surgically resectable PDAC patients is currently 44%. The late diagnosis of PDAC is a result of little or no symptoms in its early stage of development and a lack of specific biomarkers that may be utilized in routine examinations in the clinic. Although healthcare professionals understand the importance of early detection of PDAC, the research on the subject has lagged and no significant changes in the death toll of PDAC patients has been observed. This review is focused on understanding potential biomarkers that may increase the early diagnosis of PDAC patients at its surgically resectable stage. Here, we summarize the currently available biomarkers used in the clinic as well as those being developed with the hope of providing insight into the future of liquid biomarkers to be used in routine examinations for the early diagnosis of PDAC.

## 1. Introduction

Pancreatic cancer is categorized into two major groups depending on where the cancer cells originated. Most often, pancreatic cancer arises from the exocrine cancers, which include pancreatic ductal adenocarcinoma (PDAC), acinar cell carcinoma, solid pseudopapillary neoplasm and pancreatoblastoma. On the other hand, pancreatic neuroendocrine cancers, which give rise to neuroendocrine carcinomas and neuroendocrine tumors, constitute a small percentage of pancreatic cancer diagnosis.

PDAC is the most common type of pancreatic cancer, accounting for over 90% of all pancreatic cancer cases. It is also one of the most lethal types of cancer, with an extremely low five-year survival rate. While the average five-year survival rate of other types of cancer such as breast cancer, skin cancer and prostate cancer has been dramatically increased from around 40–50% in the 1970s to 90–100% currently, the five-year survival rate of pancreatic cancer has only improved from 3% to approximately 12% in the last five decades [1,2,3]. While modern technology and continuous developments in the medical field have drastically increased the average life span of human beings, it may have also resulted in an increased number of patients diagnosed with pancreatic cancer. For example, the estimated number of new cases of pancreatic cancer in the US in 1970 was 18,800, while in 2023, this number has dramatically risen to 64,050 [3,4]. Furthermore, the dismal survival rate of PDAC due to inefficient treatment options make this number more unsettling. Unfortunately, almost all patients are diagnosed with a late stage of the disease, where pancreatic cancer cells have metastasized to other organs distant from the original site, causing most PDAC patients to die within six months after clinical diagnosis [5,6]. However, if pancreatic cancer is diagnosed at an early stage such as stage 1 and 2, known as localized pancreatic cancer or resectable pancreatic cancer, the five-year survival rate can be as high as 44% in the US [7].

The late diagnosis in PDAC patients is largely due to non-descript symptoms for this disease at its early stage and a lack of regular examinations that are practical for the pancreas [8]. Therefore, it is pivotal to comprehensively understand what tools we currently have and require in order to detect PDAC at an early stage, to significantly reduce the death toll of pancreatic cancer. In this review, we summarize the currently available biomarkers and detection methods in the clinic for PDAC. Meanwhile, we also provide information on the developing biomarkers as well as insight into other potential biomarkers that may help with the early detection of localized pancreatic cancer.

## 2. Biomarkers Currently Used for PDAC in the Clinic

There are limiting screening options for individuals who are at risk of developing pancreatic cancer. Most screening options that lead to a diagnosis of pancreatic cancer are based on computerized tomography (CT) scanning, magnetic resonance imaging (MRI), and endoscopic ultrasound (EUS) biopsy [9]. These screening options can become very costly and invasive for individuals, and diagnoses of the disease are usually made after the cancer is formed within the pancreas and has metastasized to other organs, thus causing the high fatality rate of PDAC. Another option for screening for pancreatic cancer is a non-invasive procedure that detects biomarkers in blood serum samples. The FDA-approved biomarker carbohydrate antigen 19-9 (CA19-9) is currently the only widely used biomarker for pancreatic cancer diagnoses and development. This antigen is also known as Sialyl-Lewis^A^ and plays a role in cell communication [10]. The increased levels of CA19-9 suggest an advancement in pancreatic cancer; the median diagnostic sensitivity of this biomarker is 79% and the median specificity is approximately 80% [11]. CA19-9 has also proven useful as a prognostic marker for PDAC, a highly aggressive lethal malignancy that accounts for more than 90% of all pancreatic cancer cases [12,13]. The increase in the baseline of CA19-9 levels appears to be associated with poor clinical outcomes because studies have shown an increase in the sensitivity and specificity of CA19-9 with PDAC cases. The elevation in CA19-9 level has been detected as early as two years before any clinical diagnosis, providing a lead time for diagnosis of the PDAC that allows for surgical operation, thus increasing the chance of long-term survival in PDAC patients [14]. In addition, it has also been reported that CA19-9 plays a role in promoting disease pathogenesis and maintenance [15]. Therefore, targeting CA-19-9 may offer novel therapeutic options because the interruption of CA 19-9 could hinder the development and progression of PDAC. Other biomarkers that are not FDA-approved but are currently being evaluated include cancer antigen 125 (CA-125), also known as MUC16, and carcinoembryonic antigen (CEA). Independently, CA-125 has a 51% sensitivity to detecting pancreatic cancer, and when combined with CEA, the sensitivity increases to 74% for diagnosis [16].

### Reasons for the Insufficiency of Current Clinical Biomarkers for Decreasing Pancreatic Cancer Mortality

Although serum CA19-9 is known as the main biomarker for PDAC, the sensitivity for early-stage diagnosis is very low, which leads to individuals being diagnosed past the window that allows for surgical resection of PDAC [16]. Asymptomatic individuals who are diagnosed with PDAC have a 10–13% sensitivity to CA19-9 therefore, results may provide a false negative for pancreatic cancer [9,12,14]. This false negative is partially due to fucosyltransferase deficiency, which leads to a scarce amount of CA19-9 being produced, also known as Lewis antigen A negative or Lewis negative [14,16]. The Lewis gene, known as fucosyltransferase, is a key enzyme of CA19-9 biosynthesis and plays a role in protein fucosylation [17]. On the other hand, elevated levels of CA19-9, which has a correlation with bile duct obstruction, inflammation, pancreatitis, and other digestive cancers and benign conditions, may lead to a false positive for PDAC [14,16,18]. In both cases, the accuracy of the diagnosis is questionable if diagnosis is solely based on this serum biomarker. Studies have shown that cancer cells do not always secrete tumor markers or even the same tumor markers within a singular tumor; therefore, additional biomarkers are needed to accurately diagnose PDAC [11]. Some studies recommended that the three commonly used serum biomarkers—CA19-9, CA-125, and CEA—should be combined and used as a routine screening method for Lewis-negative and Lewis-positive PDAC [16]. However, these biomarkers are still ineffective as early markers for PDAC because of their low specificity [19]. Innovative circulating biomarkers are required for detecting precursor lesions of PDAC to devise screening options before or during the early stages of cancer development.

## 3. Newly Identified or Reported Biomarkers for Pancreatic Cancer

### 3.1. Diagnostic Biomarkers at Early Stages of Clinical Trails

**Figure 1:**
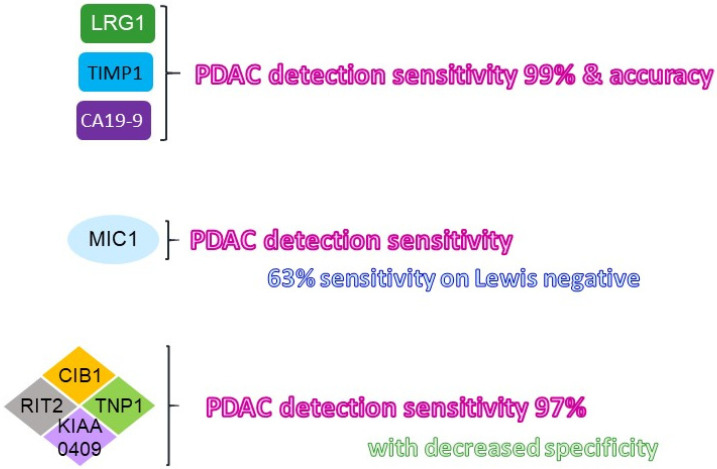
**Summary of the newly identified diagnostic liquid biomarkers for PDAC.** The newly identified liquid biomarkers for improving the sensitivity as well as the specificity of PDAC at its early stage were illustrated. The improved detection sensitivity indicated was in comparison with that using CA19-9 alone. LRG: leucine-richalpha-2 glycoprotein; TIMP: tissue inhibitor matrix metalloproteinase; MIC: macrophage inhibitory cytokine; CIB: calcium and integrin binding; TNP: transition protein; RIT2: RaslikewithoutCAAX2.

To establish novel non-invasive screening methods, researchers have identified proteins within the blood that would aid in detection of cancer cells during the early stage of PDAC. There have been many challenges with the accuracy of the early diagnosis of PDAC patients with CA19-9, due to the low expression of antigens below the detection threshold [20]. Of note, small-sized study populations and low sensitivity have prevented these newly identified biomarkers from being approved for general screening in the clinic. The expression of certain proteins, such as leucine-rich alpha-2 glycoprotein 1 (LRG1), tissue inhibitor of metalloproteinase 1 (TIMP-1), calcium and integrin binding 1 (CIB1), and macrophage inhibitory cytokine 1 (MIC1), have been identified as potential biomarkers for the early detection of pancreatic cancer (Figure 1) [14,21,22].

Both LRG1 and TIMP1 are glycoproteins that play a role in cancer progression. TIMP1 plays a crucial role in maintaining the extracellular matrix composition and wound healing, while LRG1 promotes the development of new blood vessels [23]. Previous studies using serum samples of PDAC patients who were newly diagnosed showed that the combination of LRG1, TIMP1 and CA19-9 biomarkers increased the accuracy of pancreatic cancer detection by 13.2% with a specificity greater than 99%, as compared to that using CA19-9 alone [14]. In addition, these combined biomarkers were able to blindly detect samples that were initially omitted from being positive for PDAC due to a low expression of CA19-9 from PDAC patients who were Lewis-negative.

It has been proposed that cancer-specific autoantibodies—antibodies made against substances formed by a person’s own body—could establish reliable biomarkers for the early detection of PDAC. By using an autoantigen screening system, AlphaScreen, for detecting autoantibody biomarkers in the serum samples of PDAC patients and healthy individuals, nine target proteins were upregulated in PDAC as compared to healthy individual samples [22]. Among these nine target proteins, CIB1 protein had the highest significance in PDAC diagnosis, with a value of 97.3% [22]. CIB1 protein is involved in the regulation of various cellular processes, such as cell differentiation and cell division, making it a key target for cancer treatment [24,25,26]. The combination of the other three most significant identified autoantigens, including KIAA0409, Ras like without CAAX2 (RIT2), and transition protein 1 (TNP1), with CIB1, increased the overall sensitivity to 97% [22]. However, it has also been shown that the use of these four autoantibodies led to a decrease in specificity with PDAC patients because KIAA0409, RIT2 and TNP1 were also expressed in ovarian cancer and possibly testis cancer, as well as neuronal disorders [27,28,29]. Therefore, correctly identifying PDAC using these four autoantibodies reduces specificity to only 35%, as compared to the CIB1 autoantibody alone (75.7% sensitivity and 70.0% specificity) [22].

Lastly, MIC-1 has also been shown as a potential novel biomarker in the early stages of PDAC for predicting the development and progression of the cancer [21]. MIC-1 is a secreted growth factor of the TGF-β superfamily and is upregulated in response injury, inflammation, and cancers. Studies show that increased levels of MIC-1 were present in PDAC patient serum compared to controls [21]. The sensitivity level of MIC-1 (65.1%) was higher in the earlier stages of PDAC when compared to the level of CA19-9 alone (43.0%) and combination levels of CEA and CA242 [21,30]. Furthermore, levels of MIC-1 detected in serum showed a 63.1% sensitivity with the samples that were identified as Lewis-negative, which was higher than the combined CA242 and CEA biomarkers [30].

### 3.2. Combination of Clinical Biomarkers

**Figure 2:**
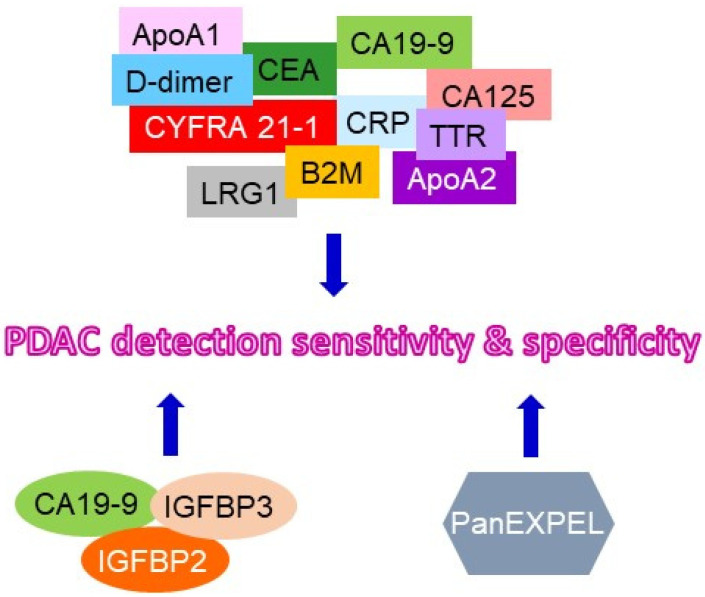
**Summary of the suggested combination of clinical liquid biomarkers for PDAC detection.** The recommended combination of clinical liquid biomarkers for improving the sensitivity and specificity of PDAC at its early stage were illustrated. ApoA1: apolipoproteinA1; CRP:C-reactive protein; CYFRA21-1: cytokeratin 19 fragment 21-1; TTR: transthyretin; LRG: leucin-richalpha-2 glycoprotein; B2M: beta-2 macroglobulin; PanEXPEL: new biomarkers in pancreatic cancer using EXPEL methodology.

As previously stated, the average five-year survival rate of PDAC is currently approximately 12%. Due to the low sensitivity of the currently approved biomarkers during the early stages of the cancer and Lewis-negative PDAC, the current research focus has been on combining multiple biomarkers to eliminate challenges associated with early diagnosis in PDAC. New biomarker combinations have been shown to increase specificity and sensitivity in PDAC patients as compared to healthy individuals (Figure 2) [11,20,31,32,33].

The combination of multiple plasma biomarkers has been used in clinical studies comparing the diagnosed values of individual plasma samples including PDAC and those of healthy individuals as a control. A biomarker panel consists of eleven candidate biomarkers (Apolipoprotein A (ApoA) 1, CA125, CA19-9, C-reactive protein, cytokeratin 19 fragment 21.1, CEA, ApoA2, transthyretin, beta-2 microglobulin, D-dimer, and LRG1) representing 2047 combinations and has shown a significant increase in specificity and sensitivity amongst PDAC samples in comparison with control samples [11]. Clinical findings also suggest that combining CA19-9 with other novel biomarkers has increased the diagnostic rate as compared to the standalone CA19-9 biomarker [11,31,32]. Levels of insulin-like growth factor-binding protein (IGFBP) 2 and IGFBP3 detected in the plasma samples have been shown to statistically separate PDAC patients from healthy individuals [20]. Therefore, a combination of IGFBP2, IGFBP3, and CA19-9 could also provide more dependable diagnoses during the early stages of PDAC.

Panels of biomarkers such as the New Biomarkers in Pancreatic Cancer using EXPEL methodology (PanEXPEL) can also provide insight into signature characteristics of the disease [33]. PanEXPEL is a database that archives the interstitial tissue fluids that are released from the lesion during diagnostic biopsy. This database provides a potential resource for identifying novel combinations of biomarkers that can be derived from proteins, metabolites, RNA, DNA, exosomes, etc., to better diagnose PDAC at early stages in the future.

### 3.3. Reported Diagnostic Biomarkers That May Move to Future Clinical Trials

**Figure 3:**
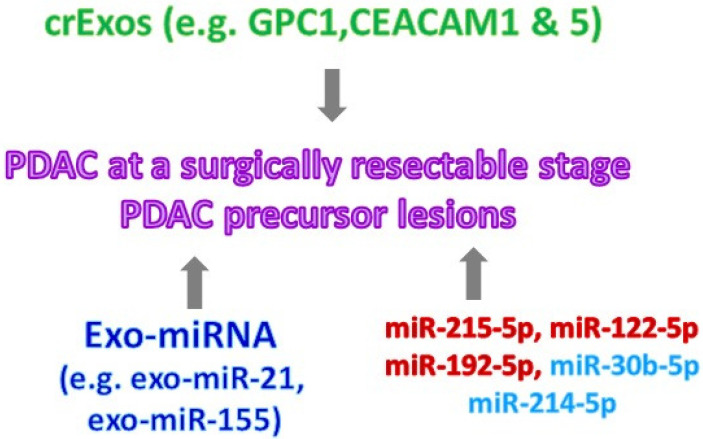
**Summary of the potential liquid biomarkers for detection of PDAC and its precursor lesions.** The reported secreted factors from PDAC and its precursor lesions which can be used as potential liquid biomarkers in the clinic were illustrated. crExos: circulating exosomes; GPC: glypican; CEACAM: carcinoembryonic antigen-related cell adhesion; exo-miRNA: exosomal microRNA; miRNA: serum microRNA.

To establish biomarkers that can be practically used in the clinic for the regular screening of early-stage PDAC, biomarkers have to be present in the liquid biopsy samples—this is known as non-invasive diagnosis. The secreted factors of PDAC, such as circulating tumor DNA, circulating tumor RNA, circulating tumor cells, and cancer exosomes, all of which are either generated as by-products or involved in PDAC progression, have been reported for their potential as biomarkers for early PDAC (Figure 3) [34,35,36,37,38]. The cell-surface proteoglycan glypican-1 (GPC1) has been identified as being specifically expressed on PDAC-derived exosomes, extracellular vesicles containing proteins, and nucleic acids [39]. A high expression of GPC1 on circulating exosomes (crExos) statistically separated serum samples between 190 PDAC patients and 100 healthy individuals. Furthermore, it has also been shown that in the presence of GPC1 on crExos in pancreatic intraepithelial neoplasia (PanIN) lesions of transgenic mice, p48^cre^; Kras^G12D^; and TGFRβII^flox/flox^ were used to recapitulate human PDAC. Of note, this study used serum samples from PDAC patients who almost all had unresectable PDAC and were diagnosed with PDAC at stage 2 and beyond. In addition to using patient serum samples [39,40], exosomal microRNAs (Exo-miRNAs) and other exosomal proteins in the pancreatic juice have also been evaluated for their potential as biomarkers for PDAC detection [35,38]. As compared to pancreatic juice collected from chronic pancreatitis patients, exo-miR-21 and exo-miR-155 were increased in PDAC patients [35]. Similarly, carcinoembryonic antigen-related cell adhesion molecules (CEACAMs), including CEACAM1 and CEACAM5, were identified on the exosomes collected from the pancreatic duct fluid of PDAC patients rather than patients with benign pancreatic diseases [38]. Similar to circulating tumor RNA and cancer exosomes, circulating tumor DNA has the potential to be a diagnostic tool for early-stage PDAC. However, the major focus in this field has been the utilization of the isolated circulating tumor DNA from PDAC patients for prognosis and assisting treatment strategies [41,42,43]. Recently, it has been reported that using the Kras mutation of the circulating tumor DNA combined with all other four protein biomarkers, including CA19-9, CEA, hepatocyte growth factor (HGF) and osteopontin (OPN), in the plasma samples of PDAC patients with stage 1 or 2 disease increased the sensitivity of PDAC detection in comparison to circulating tumor DNA alone [44]. Another study showed that using five hydroxymethylcytosine changes in several genes, such as *GATA4*, *GATA6*, *YAP1*, *TEAD1*, etc., in the isolated circulating tumor DNA from the plasma samples of PDAC or non-cancer patients may detect PDAC at an early stage [45]. Of note, in these studies, including those with patient serum samples, the major argument against them is the small sample size from PDAC patients and control/pancreatitis patients. The other limitation to this study is that pancreatic duct fluid, known as pancreatic juice samples, are not samples that can be obtained from a common checkup, which further limits their practical use in the clinic for the purpose of PDAC early diagnosis. Fortunately, other early-detection methods using miRNA have been shown as promising diagnostic tools.

Recently, a panel of serum miRNAs has been suggested specifically for diagnosing and detecting PDAC early [46]. Eight circulating miRNAs, including miR-215-5p, miR-122-5p, miR-192-5p, miR-181a-2-3p, miR-30b-5p, miR-216b-5p, miR-320b and miR-214-5p, were detected by real time qRT-PCR in the serum samples of 50 PDAC and 50 chronic pancreatitis patients and 25 healthy individuals. Five out of these eight miRNAs were increase miR-215-5p, miR-122-5p and miR-192-5p, and there was a decrease in miR-30b-5p and miR-320b in PDAC serum, suggesting a potential role for a miRNA panel containing these five specific miRNAs for diagnosing PDAC early [46]. A larger cohort of PDAC patients who have resectable PDAC is required to further validate the possible use of this miRNA panel for the early detection of PDAC in the clinic.

Although several biomarkers that can be detected in the liquid biopsy samples of PDAC patients have been identified, as described above, the main concern is that the majority of the liquid biopsy samples in these reported studies were from PDAC patients who had an unresectable disease. The other concern is the small sample size of the cohorts used in these studies. Because of these concerns, it remains unclear whether these reported biomarkers can be used to diagnose resectable PDAC (stage 1) early in the clinic for high-risk populations, such as people with familial pancreatitis or diabetes and the senior population [47,48,49,50,51].

## 4. Biomarkers for Pancreatic Cancer Precursors

PanIN, intraductal papillary mucinous neoplasm (IPMN), and mucinous cystic neoplasm (MCN) are precursors of PDAC. PanIN is derived from acinar cells transdifferentiating to a highly proliferative duct-like phenotype, known as acinar-to-ductal metaplasia (ADM). Given that 85–95% of PDAC patients [52] are diagnosed with an unresectable disease because cancer cells are often too close to arteries, veins, and lymph nodes (local advanced PDAC) or have already metastasized outside of the pancreas to other organs (metastatic PDAC), the development of biomarkers for these PDAC precursors will not only allow for early detection but will also provide better strategies for treatments and interventions for this deadly disease.

Although compiling evidence showed how pancreatic ADM is regulated, the majority of the reported ADM regulators are transcription factors [53,54,55,56,57,58], which makes them inadequate liquid biomarkers for routine use in liquid biopsy samples. Interestingly, some secreted factors/proteins involved in inflammation and lipid metabolism have been shown to promote the pancreatic ADM process [58,59,60,61]. It has been demonstrated that treating primary murine pancreatic acini with recombinant Reg3A, a secreted C-type lectin that can function as growth factors, opsonins, antimicrobial proteins and components of the extracellular matrix (ECM), induced ADM in a 3D organoid culture [59]. In addition to Reg3A, macrophage-secreted cytokines, including tumor necrosis factor (TNF) and RANTES, also have been shown to drive pancreatic acini transdifferentiation to a duct-like phenotype [58,60]. In a 3D organoid culture system of primary murine pancreatic acini, the neutralization of TNF or RANTES via their specific neutralizing antibodies reduced macrophage condition media-mediated pancreatic ADM [38]. Furthermore, exogenously added recombinant TNF or RANTES resulted in the transdifferentiation of primary acini to duct-like cells. The expression of macrophage-secreted TNF and RATES was detected in the human ADM regions of the pancreas. It also has been shown that TNF is able to not only increase Kras-mediated pancreatic ADM events but also further enhance the size of the formed duct-like structures in the 3D organoid culture [60], supporting the idea that TNF and RANTES are suitable as liquid biopsy markers for PDAC precursors. Angiopoietin-like 4 (ANGPTL4), a secreted protein modulating triacylglycerol homeostasis [62], has been reported to expediate oncogenic Kras^G12D^-mediated ADM of the pancreas via the activation of periostin [61]. Furthermore, levels of the ANGPTL4 protein were expressed in human and mouse ADM regions of the pancreas, thus implying a different cellular localization of ANGPTL4 rather than its secretion. Whether ANGPTL4 can be utilized as one of the liquid biomarkers for ADM lesions requires further investigation.

Based on the findings from transgenic mouse models of PDAC, PanIN cells that harbor only certain gene mutations, such as *INK4A* and *TP53,* in a specific sequence can eventually give rise to PDAC. However, in a recent study, using laser-capture microdissection coupled with whole-exome sequencing technology to analyze PanIN cells at different stages of progression from PDAC patients, researchers showed that PDAC patients within the same stage of disease progression had various PanIN gene mutations. These results suggest that PanIN cells, the precursors for PDAC, are able to migrate and clonally expand to form one or more PanINs in the pancreatic ductal system in the early stages of development [63]. This study shows that it may be possible to use these migratory PanIN cells in the liquid biopsy samples as a biomarker for PDAC at its precursor stage. In addition to the disseminated PanIN cells, research has recently focused on identifying other secreted factors, including, but not limited to, exosomes released from PDAC precursors as biomarkers for the early diagnosis of pancreatic tumors progressing into resectable PDAC [64,65]. Cell migration-inducing protein (CEMIP), also known as KIAA1199, involved in the depolymerization of hyaluronic acid in the extracellular matrix, has been detected in the serum samples of pdx1^cre^; Kras^G12D^ transgenic mice that harbored PanIN lesions [64]. However, it is unclear if CEMIP is secreted by human PanIN lesions to the bloodstream or any other types of liquid biopsy samples. As for IPMN lesions, it has been reported that the high expression of MUC5AC from crExos in the serum samples separated patients who had invasive IPMNs from these with low-grade IPMN lesions [65]. Altogether, these studies shed light on the identification of more liquid biomarkers specific for PDAC precursors. With further advancement in this field, it is anticipated that a panel of a combinational secreted factors released by high-grade PDAC precursor lesions will be established to diagnose these tumors early, before they become unresectable PDAC.

## 5. Conclusions

PDAC accounts for the majority of pancreatic cancer cases with a fatal prognosis. The importance of the early detection of pancreatic cancer has forced researchers to develop solutions for patients and individuals at risk of developing pancreatic cancer. Unfortunately, the current protocol—screening for serum-based and other liquid biomarkers in the clinic—has resulted in negligible success as it pertains to the diagnosis and prognosis of PDAC. Currently, there is only one FDA-approved serum biomarker—CA19-9—for pancreatic cancer (See Table 1 for a summary of the biomarkers mentioned in this review). However, the low sensitivity of this marker during the early stages of PDAC often leads to misdiagnosis or late-stage diagnosis. Ongoing clinical studies suggest that a panel of biomarkers that is comprised of proteins such as CA125, CIB1, MIC-1, RIT2, TNP1, and KIAA0409 (to increase sensitivity), along with CA19-9, a specific biomarker of PDAC, may lead to earlier diagnosis and a significant increase in the overall survival rate. Meanwhile, studies encompassing other biomolecule secreting factors, such as circulating tumor DNA, circulating tumor RNA, circulating tumor cells, and cancer exosomes, have also shown their potential as early detectors of PDAC. Although these studies have not yet been confirmed for practical use, they also suggested the possibility of biomarkers within the liquid biopsy. For example, miRNAs and migratory PanIN cells could be used for the early detection of PDAC while PDAC is still in its surgically resectable stage. A panel of biomarkers that are sensitive to Lewis-negative individuals, such as MIC-1 and CA125, along with PDAC-specific marker CA19-9, may also make surgical resection possible for the majority of PDAC patients through early detection in the future. Although numerous potential liquid biomarkers have been reported, as described in this review (Table 1), there are several limiting factors for the use of these biomarkers for the early detection of PDAC. One of the biggest limitations is that the majority of these reported liquid biomarkers were tested in PDAC patients who had an unresectable disease. It remains unclear if any of these liquid biomarkers are capable of detecting PDAC at its early stage (surgically resectable stage) and whether they can be used as a diagnostic tool. Screening tools for PDAC are typically costly and not covered by many health insurance policies, and therefore they are only implemented in cases of individuals who are at high risk for PDAC. This screening strategy has failed to reduce the death-toll number of individuals diagnosed with PDAC. To continue the advancement of developing novel screening tools to diagnose PDAC at its surgically resectable stage, research studies must focus on liquid biomarkers for pancreatic tumors and PDAC precursor lesions that may be found in annual blood tests.

## Figures and Tables

**Table 1:** Summary of the used or reported biomarkers for PDAC and its precursor lesions.

Biomarkers	FDA Approval	Disease	Specificity	Sensitivity	Fluid Type	Reference
CA19-9	Yes	Pancreatic Cancer (Symptomatic)	79%	80%	Blood/serum	[12]
CA19-9	Yes	Pancreatic Cancer (Asymptomatic)	ND*	13%	Blood/serum	[9,12,14]
CA19-9	Yes	PDAC (Symptomatic)	ND*	43%	Blood/serum	[18,21]
CA125 + CEA	No	PDAC	ND*	74%	Blood/serum	[16]
CA125	No	PDAC	ND*	51%	Blood/serum	[16]
LRG1 + TIMP1 + CA19-9	No	PDAC	>99%	ND*	Blood/serum	[14]
KIAA0409 + RIT2 + TNP1 + CIB1	No	PDAC	35%	97%	Blood/serum	[22]
CIB1	No	PDAC	70%	76%	Blood/serum	[22]
MIC-1	No	PDAC (Symptomatic)	ND*	65%	Blood/serum	[18,21]
MIC-1	No	PDAC (Asymptomatic)	ND*	63%	Blood/serum	[21]
MIC-1 + CA19-9	No	PDAC	ND*	78%	Blood/serum	[30]
ApoA1 + CA-125 + CA19-9 + CEA + CA19-9 + D-Dimer + CYFRA 21-1 + TTR + ApoA2 + B2M + LRG1	No	PDAC	ND*	ND*	Blood/plasma	[11]
Circulating tumor DNA: *Kras* mutation + CA19-9 + CEA + HGF + OPN	No	PDAC	ND*	64%	Blood/plasma	[44]
*Kras* mutation + 5 hydroxymethylcytosine modification in *GATA4, GATA6, YAP1, TEAD1*	No	PDAC	ND*	ND*	Blood/plasma	[45]
Circulating tumor cells	No	PDAC	ND*	ND*	Blood/serum	[34,35,36]
Circulating tumor RNA: miR-215-5p, miR-122-5p, miR-192-5p, miR-181a-2-3p, miR-30b-5p, miR-216b-5p, miR-320b and miR-214-5p	No	PDAC and its precursor lesions	ND*	ND*	Blood/serum	[41]
Cancer exosomes: GPC1 and Exo-miRNA	No	PDAC and its precursor lesions	ND*	ND*	Pancreatic juice	[35]
CEACAM1 and 5		PDAC and its precursor lesions	ND*	ND*	Pancreatic juice	[38]
Migratory PanIN cells	No	PDAC precursor lesions	ND*	ND*	NA^#^	[58]
TNF	No	Early event to initiate PDAC	ND*	ND*	NA	[53,55]
RANTES	No	Early event to initiate PDAC	ND*	ND*	NA	[53,55]

ND*: not determined nor mentioned by the publication; NA^#^: not applicable since the study only used tissue samples instead of any bioliquid samples; NA: not applicable since the experiments are only in vitro.

## Data Availability

Not applicable.
